# Refinement of inducible gene deletion in embryos of pregnant mice

**DOI:** 10.1002/bdr2.1628

**Published:** 2019-12-03

**Authors:** Dawn Savery, Eirini Maniou, Lucy H. Culshaw, Nicholas D. E. Greene, Andrew J. Copp, Gabriel L. Galea

**Affiliations:** ^1^ Developmental Biology and Cancer UCL GOS Institute of Child Health London UK; ^2^ Comparative Bioveterinary Sciences Royal Veterinary College London UK

**Keywords:** 3Rs, CreERT2, mouse, Tamoxifen, TAT‐Cre

## Abstract

CreERT2‐mediated gene recombination is widely applied in developmental biology research. Activation of CreERT2 is typically achieved by injection of tamoxifen in an oily vehicle into the peritoneal cavity of mid‐gestation pregnant mice. This can be technically challenging and adversely impacts welfare. Here we characterize three refinements to this technique: Pipette feeding (not gavage) of tamoxifen, ex vivo CreERT2 activation in whole embryo culture and injection of cell‐permeable TAT‐Cre into Cre‐negative cultured embryos. We demonstrate that pipette feeding of tamoxifen solution to the mother on various days of gestation reliably activates embryonic CreERT2, illustrated here using *β‐Actin* ^*CreERT2*^, *Sox2* ^*CreERT2*^, *T* ^*CreERT2*^, and *Nkx1.2* ^*CreERT2*^. Pipette feeding of tamoxifen induces dose‐dependent recombination of *Rosa26* ^*mTmG*^ reporters when administered at E8.5. Activation of two neuromesodermal progenitor‐targeting Cre drivers, *T* ^*CreERT2*^, and *Nkx1.2* ^*CreERT2*^, produces comparable neuroepithelial lineage tracing. Dose‐dependent CreERT2 activation can also be achieved by brief exposure to 4OH‐tamoxifen in whole embryo culture, allowing temporal control of gene deletion and eliminating the need to treat pregnant mice. *Rosa26* ^*mTmG*^ reporter recombination can also be achieved regionally by injecting TAT‐Cre into embryonic tissues at the start of culture. This allows greater spatial control over Cre activation than can typically be achieved with endogenous CreERT2, for example by injecting TAT‐Cre on one side of the midline. We hope that our description and application of these techniques will stimulate refinement of experimental methods involving CreERT2 activation for gene deletion and lineage tracing studies. Improved temporal (ex vivo treatment) and spatial (TAT‐Cre injection) control of recombination will also allow previously intractable questions to be addressed.

## 1 INTRODUCTION

Induction of conditional gene deletion through tamoxifen‐mediated activation of a Cre recombinase enzyme linked to a mutated estrogen receptor domain, CreERT2, is a mainstay technique in developmental biology research. Administered tamoxifen base is hepatically metabolized to produce various biologically active metabolites including 4OH‐tamoxifen (OHT). OHT binds the mutated estrogen receptor domain of CreERT2 and causes it to translocate to the nucleus, where it recombines floxed alleles, enabling genes of interest to be deleted (Brocard et al., 1997; Felker et al., 2016). Tamoxifen metabolites exert biological activities as estrogen receptor modulators beyond their activation of CreERT2, such as inducing new bone formation (Dutertre & Smith, 2000; Sugiyama, Galea, Lanyon, & Price, 2010). These off‐target effects are particularly problematical in developmental biology research as they cause embryo toxicity, loss of pregnancy and inability to induce parturition (Lee, Lee, Park, Zhang, & Jin, 2012; Lizen, Claus, Jeannotte, Rijli, & Gofflot, 2015; Park et al., 2008; Pugh & Sumano, 1982; Ved, Curran, Ashcroft, & Sparrow, 2019). Nonetheless, we and others commonly inject tamoxifen dissolved in an oil‐based vehicle intraperitoneally (IP) into the abdomen of mid‐gestation pregnant mice in order to induce CreERT2 recombination in their embryos. IP injection is not recommended in gravid animals due to the increased risk of misinjection (Morton et al., 2001). Nonetheless, this approach has been tremendously valuable in testing the effects of embryonic gene deletion and lineage tracing cell populations.

The mode of tamoxifen administration varies depending on the desired endpoint. These include IP or subcutaneous (SC) injections, implantation of osmotic pumps, gavage and medicated diets. Human patients take tamoxifen as oral tablets. Mice also absorb tamoxifen following oral dosing and convert it to OHT (Reid et al., 2014). While the bioavailability of the tamoxifen parent compound is lower for oral than SC administration, OHT reaches a higher *C*_*Max*_ with a shorter *T* ^*1/2*^ following oral than SC dosing (Reid et al., 2014). A shorter OHT *T* ^*1/2*^ is typically preferable for embryological studies as it provides greater temporal control over gene deletion. Oral gavage of tamoxifen has previously been used in developmental biology research, for example to trace cells derived from the *Nkx1.2* ^*CreERT2*^‐expressing lineage of neuromesodermal progenitors (NMPs) (Rodrigo Albors, Halley, & Storey, 2018). A previous study observed less embryotoxicity following gavage rather than IP tamoxifen administration (Park et al., 2008). However, gavage also has the potential to substantially impair mouse welfare: a study of repeated gavage tolerability in mice reported the technique was generally well tolerated, but two out of 13 gavaged mice died with thoracic pathology during the study (Arantes‐Rodrigues et al., 2012). Maternal stress is well known to cause embryonic loss and teratogenicity, including neural tube defects (Chernoff, Miller, Rosen, & Mattscheck, 1988; Wiebold, Stanfield, Becker, & Hillers, 1986).

In the course of our research into normal and pathological neural tube development, we sought to optimize CreERT2 activation in order to maximize maternal mouse welfare while also increasing spatial and temporal control over gene deletion. In this report, we describe three techniques adapted to achieve these goals. The first is tamoxifen administration by feeding the mother tamoxifen dissolved in oil using a pipette directly into her mouth, not by gavage. The second is ex vivo activation of CreERT2 using brief exposure to OHT in whole embryo culture. The third is local Cre administration by injecting a commercially available cell‐permeable TAT‐Cre construct. In each case, recombination is shown using the induction of lineage‐tracing reporter gene expression. We hope future studies requiring CreERT2 activation will adapt these approaches for their specific purposes, to the exclusion of more invasive methods, thereby reducing and refining their use of mice.

## 2 MATERIALS AND METHODS

### 2.1 Reagents and solutions

Tamoxifen base (Sigma, T5648‐1G) was dissolved in peanut oil to a final concentration of 100 mg/ml. This is the maximum concentration of tamoxifen, which is practically achievable without precipitation, reducing the volume that needs to be administered. Tamoxifen powder was collected at the bottom of a 15 ml conical tube using 10% v/v ethanol. Peanut oil (Sigma, P2144‐250ML) preheated to 56°C was then added and vortexed vigorously. The suspension was incubated in the dark at 56°C for 5 to 8 h with periodic vigorous vortexing. Once dissolved, the resulting solution was aliquoted into 120 μl amounts and stored at ‐20°C in the dark until use. Each aliquot was only freeze‐thawed once. The aliquot needed to be thawed directly at 56°C for a minimum of 10 minutes immediately prior to use in order to avoid precipitation. When working with sensitive CreERT2 drivers it is easier to dissolve tamoxifen at lower stock concentrations and administer up to 100 μl to an adult mouse (weighing ~20 g).

OHT was purchased from Sigma (579002‐5MG), dissolved in ethanol to a stock concentration of 10 mM and stored at –20°C for up to 1 month before use.

Cell‐permeable TAT‐Cre (Nolden et al., 2006) was purchased from Merck Millipore (SCR508) and stored in 5 μl aliquots at ‐20°C.

Rat serum for whole embryo culture was produced in house as previously described (Culshaw, Savery, Greene, & Copp, 2019; Pryor, Massa, Savery, Greene, & Copp, 2012).

### 2.2 In vivo studies and whole embryo culture

Studies were performed under the UK Animals (Scientific Procedures) Act 1986 and the Medical Research Council's Responsibility in the Use of Animals for Medical Research (1993). Mice were maintained on a 12 hourlight:dark cycle at 21+/‐1oC and provided standard lab chow and water ad libitum. Female mice were housed in groups of up to five mice, stud males were individually housed in individually ventilated, specified pathogen free cages with absorbent bedding and cardboard tube. Mice were time‐mated overnight and the morning a copulation plug was identified was considered E0.5. All animals were maintained as inbred colonies bred in‐house. Male mice were Cre positive and no adverse phenotypes were observed in adult mice used for these studies. *Rosa26* ^*YFP*^ and mTmG reporter mice were as described (Galea et al., 2017; Muzumdar, Tasic, Miyamichi, Li, & Luo, 2007; Rolo et al., 2016). *β‐Actin* ^*CreERT2*^ *, SRY‐Box (Sox)2* ^*CreERT2*^ *, brachyury (T)* ^*CreERT2*^ *and NK1 Homeobox 2 (Nkx1.2)* ^*CreERT2*^ were as previously described (Anderson et al., 2013; Andoniadou et al., 2013; Galea et al., 2017; Rodrigo Albors et al., 2018) and embryos analyzed were heterozygous for CreERT2. In each case, Cre‐negative female mice were bred with CreERT2‐expressing males overnight. Cre‐negative littermate controls from C57Bl/6 background colonies were used as wild‐type controls. Females were mated when ~8–12 weeks old (weighing ~20 g). The morning after a plug was identified was considered E0.5. At the desired stage of gestation the female mouse was loosely scruffed, such that they were able to move their head sufficiently to swallow normally, and held vertically as tamoxifen solution was slowly pipetted as small drops next to the diastema using a Gilson P200 pipette attached to an appropriate disposable tip. Mice generally ate these drops. Cutting the last 1–2 mm of the disposable pipette tip aids administration and ensures the tip end is not sharp.

Whole embryo roller bottle culture was performed as previously described (Culshaw et al., 2019; Pryor et al., 2012). OHT treatment was achieved by culturing dissected embryos in 0.3 ml/embryo rat serum containing the desired concentration of OHT for 1 h. Embryos were then washed three times by briefly passing them through DMEM with 10% FBS preincubated at 37°C, before placing them back into fresh rat serum without OHT and cultured for 24 h. Viability was judged at the end of culture or after dissection from the uterus based on the presence of a strong heartbeat, lack of pericardial or subcutaneous oedema and general morphology.

TAT‐Cre was injected by mouth pipetting, which was performed as previously described (Pryor et al., 2012). Immediately prior to use, TAT‐Cre solution was mixed in a 1:1 ratio with CellMask™ Deep Red (ThermoFisher, C10046) to aid visualization while injecting into embryonic tissue. While Fast Green is often used to visualize injection, the low pH of this solution might impair TAT‐Cre activity and so it was not used for these studies. CellMask regularly aggregates at the tip of the mouth pipette capillary and needs to be cleared between embryos. In order to achieve unilateral recombination, the capillary was inserted through the distal tip of the neural plate parallel to a neural fold and TAT‐Cre was injected as the needle was retracted.

### 2.3 Microscopy

Fluorescent stereoscope images were taken on a Leica MZFLIII microscope with an IDS UI‐1240SE camera.

Wholemount confocal fluorescent images were captured on a Zeiss Examiner LSM880 confocal using a 20x/NA1.0 or 10x/NA0.5 Plan Apochromat dipping objectives as previously described (Butler et al., 2019; Galea et al., 2017). Embryos were typically imaged with X/Y pixel sizes of 0.59 μm (speed = 8, bidirectional imaging, 1,024 × 1,024 pixels). Images were processed with Zen2.3 software and visualized as maximum projections in Fiji.

### 2.4 Statistical analysis

The majority of endpoints were qualitative (reporter gene visualization) and were observed in the embryos from at least three mice treated independently. Proportions were compared with X^2^ tests. Means between multiple groups were compared using ANOVA with post‐hoc Bonferroni. *p* < .05 was considered significant. For experiments in which the embryo phenotypes were analysed, the embryo was the unit of measure and the number of equivalently‐treated litters these embryos were derived from is specified in the figure legend. When assessing pregnancy rate, the mouse was the unit of measure. For dose‐response experiments, litters were treated sequentially with each dose until at least two litters with Cre‐positive embryos were collected at each treatment dose. Unless otherwise stated, treatment responses between different doses/time points were too obvious for meaningful blinding.

## 3 RESULTS

### 3.1 Pipette‐feeding tamoxifen robustly activates various CreERT2 drivers relevant to developmental biology research

**Figure 1:**
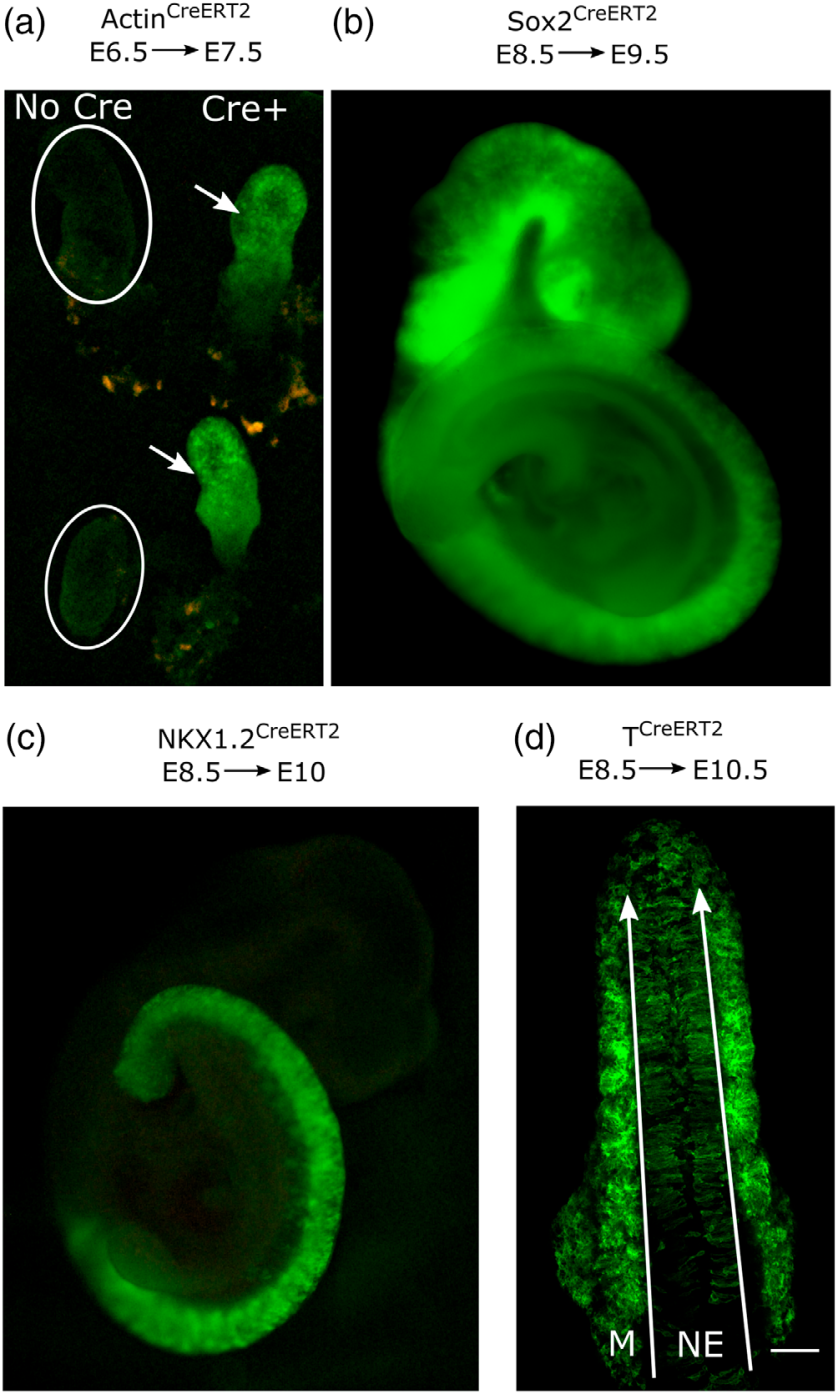
Pipette feeding of tamoxifen to pregnant female mice activates various embryonic CreERT2 drivers relevant for developmental biology research. (a–d) CreERT‐negative female mice were lightly scruffed and fed a single dose of 75 μl peanut oil containing 100 mg/ml tamoxifen free base. Recombination of *Rosa26* ^*YFP*^ (b) or *Rosa26* ^*mTmG*^ (a, c, d) reporters, producing green fluorescence. (a) Stereoscope image of *Actin* ^*CreERT2*^‐positive (arrows) and negative (circles) embryos collected at E7.5, 24 h after tamoxifen administration. (b) Stereoscope image showing extensive *Sox2* ^*CreERT2*^ recombination in an embryo collected at E8.5, 24 h after tamoxifen. (c) Stereoscope image showing *Nkx1.2* ^*CreERT2*^ recombination in an embryo collected at E10, 36 h after tamoxifen. (d) Confocal image of a dorsal view of the caudal end of a *T* ^*CreERT2*^–expressing embryo showing lineage tracing of both neuroepithelial (NE) and mesodermal (M) populations. Long arrows indicate the tail end in the confocal image; scale bar = 100 μm

All adult female mice administered tamoxifen orally by pipette feeding tolerated this with minimal agitation. Tamoxifen administration did not alter the proportion of females found not to be pregnant at the experimental endpoint; 10% of the 67 mice administered tamoxifen were found not to be pregnant at the required experimental time‐point, compared with 14% of 273 female mice collected over the same period for related experiments not requiring tamoxifen administration (*X* ^*2*^ *p* = .50). A single oral dose of tamoxifen at E6.5 robustly induced *Rosa26* ^*YFP*^ recombination by *β‐Actin* ^*CreERT2*^ producing extensive recombination throughout the embryo at E7.5 (Figure 1a). Oral tamoxifen similarly induced activation of *Sox2* ^*CreERT2*^, producing reporter gene recombination throughout the closed neural tube 24 h later (Figure 1b). Persistent lineage tracing following oral tamoxifen was also tested using two presumptive markers of the NMP population. Both *Nkx1.2* ^*CreERT2*^ and *T* ^*CreERT2*^ continued to lineage‐trace neuroepithelial and mesodermal cells 44–48 h after a single dose of oral tamoxifen (Figure 1c,d). As reported for activation of these CreERT2 drivers following IP injection of tamoxifen, these studies demonstrate oral administration of tamoxifen to the pregnant female induces embryonic CreERT2 activation at various stages of development and using a range of CreERT2 drivers.

**Figure 2:**
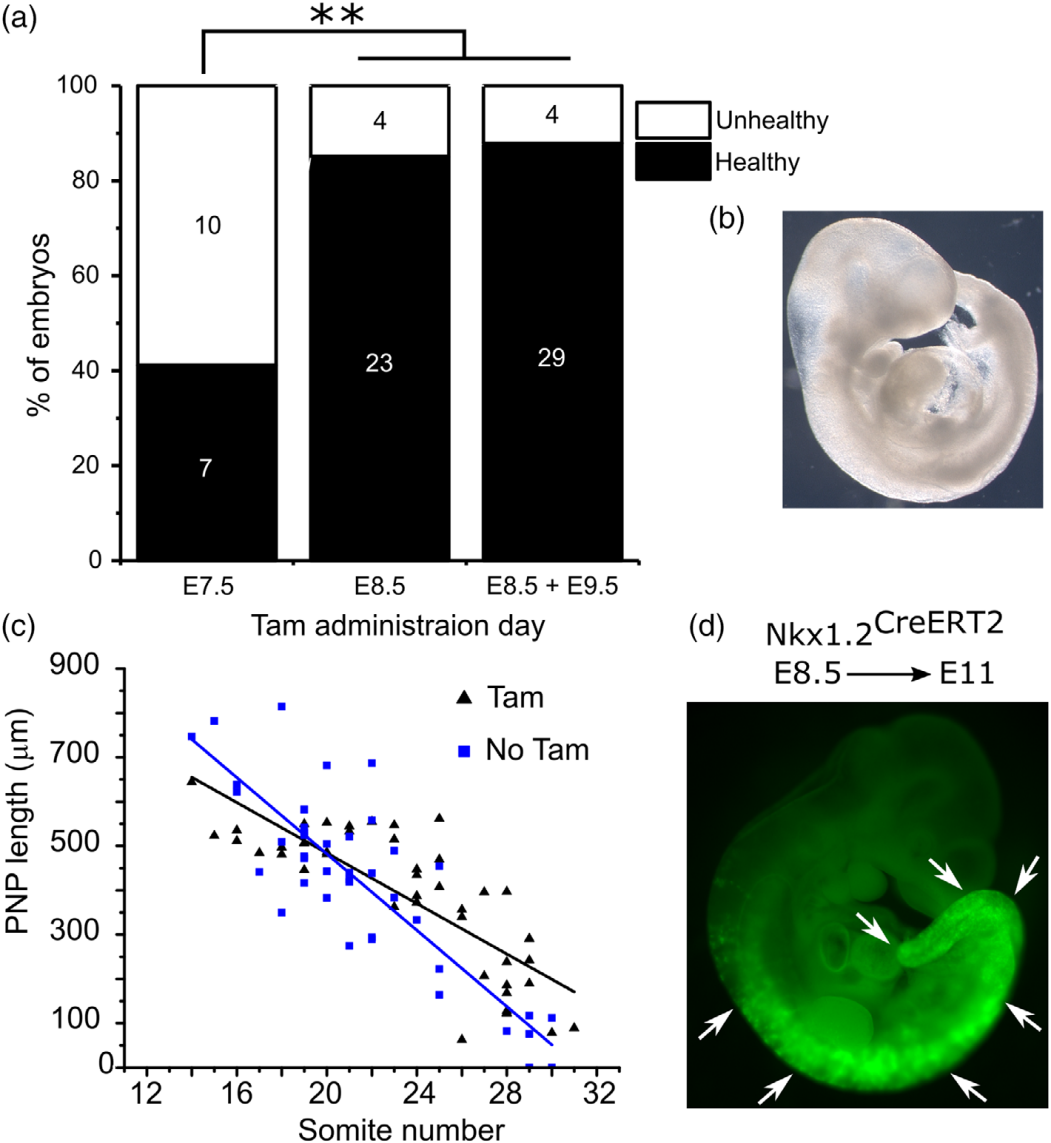
Tamoxifen administration is more toxic to embryos when administered at E7.5 than E8.5/E9.5. (a) Cre‐negative pregnant mice were pipette‐fed 7.5 mg tamoxifen on E7.5 (two litters due to the obvious toxic effects on multiple embryos in each litter), E8.5 (three litters) or both E8.5 and E9.5 (three litters). Litters were collected at E11 to assess viability. N numbers are indicated on the bars. (b) Representative “unhealthy” embryo (note over‐expanded brain and pericardial oedema) from a litter exposed to tamoxifen at E7.5. (c) PNP length of E9.5 embryos at the indicated somite stages from litters exposed to 10 mg/mouse tamoxifen on E8.5 (Tam) and litters not exposed to tamoxifen (collected in the course of related research). PNP lengths were measured blinded to somite stage. (d) Healthy embryo from a litter exposed to tamoxifen at E8.5 showing continued growth indicated by lineage tracing of *Nkx1.2* ^*CreERT2*^ along the body axis. ** *p* < .01

IP injection of tamoxifen at early stages of gestation is known to be embryotoxic (Ved et al., 2019). In order to more thoroughly characterize pipette feeding of tamoxifen, we initially determined embryo viability. Administration of a high dose of tamoxifen, 10 mg/mouse, at E7.5 was overtly toxic to ~60% of embryos recovered at E11 (Figure 2a,b, e.g., note stunted growth and pericardial oedema in Figure 2b). Administration of the same, single dose 24 h later, at E8.5, or of two doses at E8.5 and E9.5, produced significantly fewer unhealthy embryos at E11 (Figure 2a). Oral administration of 10 mg/mouse tamoxifen on E8.5 had no discernible effect on closure of the neural tube in C57Bl/6‐background embryos: 100% of 26 Cre‐negative embryos collected 48 h after tamoxifen had fully closed their neural tube. Developmental stage‐related shortening of the open spinal neural tube, the posterior neuropore (PNP), was equivalent to embryos in untreated litters (Figure 2c). Continued embryo growth is shown by Nkx1.2^CreERT2^ lineage tracing along the body axis (Figure 2d).

**Figure 3:**
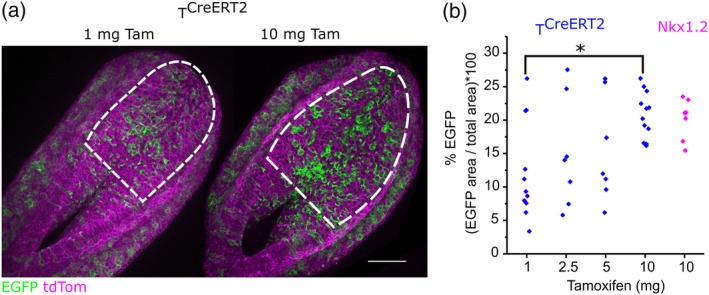
Pipette feeding of tamoxifen can induce CreERT2 activation dose‐dependently. (a) Representative confocal images showing T^CreERT2^ lineage tracing in a subset of neuroepithelial and mesodermal cells 24 h following administration of 1 mg or 10 mg tamoxifen to the mother. Dashed lines indicate the regions quantified in B, scale bar = 100 μm. (b) CreERT2 was activated by pipette‐fed tamoxifen in neuromesodermal progenitor cells, which lineage trace a subset of PNP neuroepithelial cells, using two lineage‐selective Cre drivers 24 h before embryos were collected. *T* ^*CreERT2*^ was activated with the indicated doses and *Nkx1.2* ^*CreERT2*^ produced comparable recombination when activated with 10 mg tamoxifen. * *p* < .05, each point represents an individual embryo (1 mg and 10 mg were from four litters, 2.5 mg and 5 mg were from 2 litters).

The potential to use oral tamoxifen administration to dose‐dependently induce gene recombination in different proportions of cells was tested by feeding pregnant mice different quantities of tamoxifen and assessing neuroepithelial *T* ^*CreERT2*^ lineage tracing in their embryos. While low doses of tamoxifen tended to produce variable levels of recombination, 10 mg/mouse tamoxifen caused recombination in a significantly greater proportion of the neuroepithelium than 1 mg/mouse (Figure 3a,b). *Nkx1.2* ^*CreERT2*^ and *T* ^*CreERT2*^ produced indistinguishable levels of recombination in the neuroepithelium 24 h after tamoxifen administration (Figure 3b).

### 3.2 Brief OHT exposure in whole embryo culture induces CreERT2 activation

**Figure 4:**
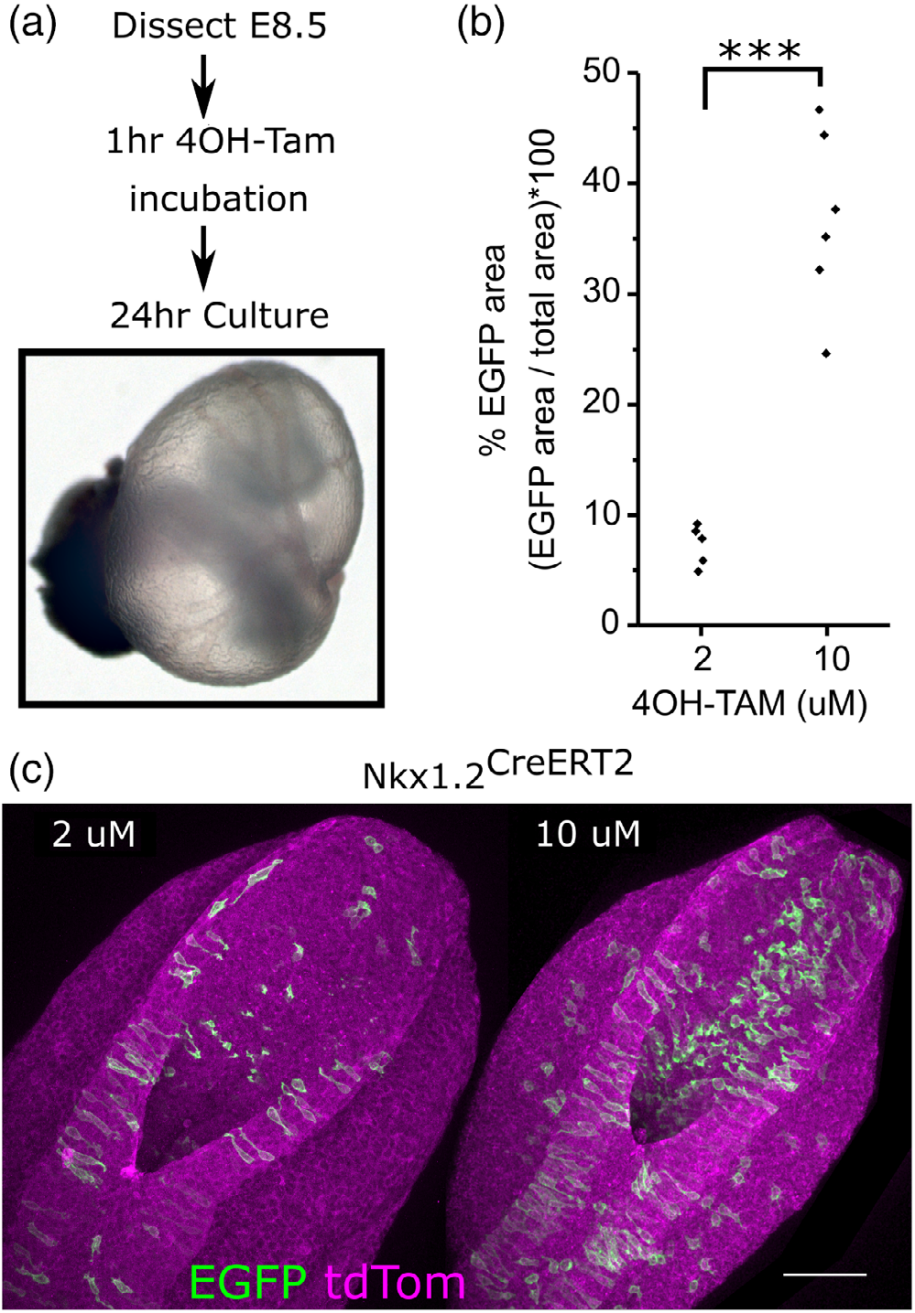
CreERT2 activation can be temporally controlled by brief exposure to 4OH‐tamoxifen in whole embryo culture. (a) Schematic of the protocol used and representative embryo at the end of culture showing an inflated yolk sac and well‐formed yolk sac vasculature. (b) Quantification of PNP recombination in *Nkx1.2* ^*CreERT2*^‐expressing mTmG embryos treated for 1 h with either 2 or 10 μM 4OH‐tamoxifen. (c) Representative images of embryos quantified in (b). Scale bar = 100 μm, *** *p* < .001, each point represents an individual embryo from three independent cultures at each concentration

While the *T* ^*1/2*^ of OHT is shorter following oral than SC injection (Reid et al., 2014), it is often desirable to time CreERT2 activation more precisely in order to test short‐term recombination effects or determine protein stability. Initial attempts to culture embryos in the presence OHT for prolonged periods of time impaired development in culture (not shown). In contrast, embryos developed normally in culture when only exposed to OHT for 1 h (Figure 4a). One hour exposure to 10 μM OHT produced significantly more recombination than 2 μM (Figure 4b,c).

### 3.3 TAT‐Cre injection permits spatial control of recombination

**Figure 5:**
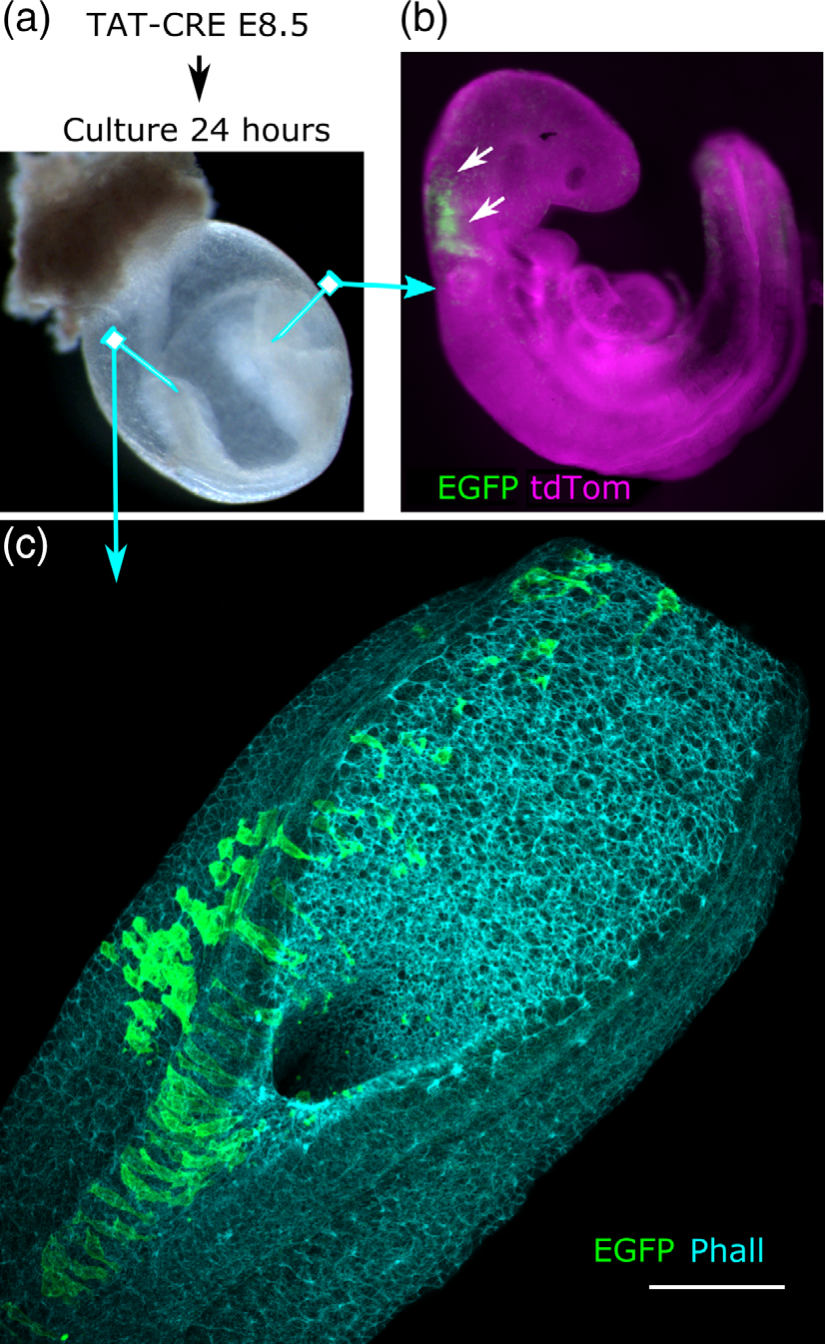
Spatially restricted gene recombination achieved by injecting TAT‐Cre. (a) Schematic of the experimental protocol. Arrows indicate the locations of TAT‐Cre injection by mouth pipetting (b) transversally into a cranial neural fold or (c) in a rostrocaudal line along a posterior neural fold 24 h prior to collection of the embryos. (b) Stereoscope image of a Cre‐negative *Rosa26* ^*mTmG*^ embryo image showing regional recombination in the hindbrain 24 h following local TAT‐Cre injection. (c) Confocal image of a PNP showing regional recombination 24 h following TAT‐Cre injection into one neural fold. Phall, phalloidin; scale bar = 100 μm

While CreERT2 activation permits lineage‐specific gene deletion, it nonetheless necessitates tamoxifen administration at a time point when the CreERT2 is robustly expressed and cannot be restricted to subsets of Cre‐ERT2‐expressing cells. It is possible to circumvent this in cultured cells by applying a cell‐permeable TAT‐Cre, deleting floxed alleles without requiring genetic expression of a Cre allele (Nolden et al., 2006). Here we demonstrate that this technology is also applicable to developmental biology research through injection in whole embryo culture (Figure 5a). Preliminary attempts to inject TAT‐Cre diffusely into the amniotic cavity produced minimal scattered recombination (negative data not shown). However, direct injection into tissues produces clear regional recombination 24 h after injection (Figure 5b). Refinement of the injection technique enables targeting of recombination to one half of the embryonic midline (Figure 5c).

## 4 DISCUSSION

Inducible gene recombination most commonly seeks to achieve one of two aims: lineage‐trace CreERT2‐labeled populations or test the effects of changing gene expression. The efficiency of gene recombination by CreERT2 in embryos is often limiting, necessitating repeated injections with tamoxifen into the abdomen of pregnant mice. Tamoxifen not only causes embryotoxicity (Ved et al., 2019), but its injection cumulatively compromises welfare. Here we show that pipette‐feeding tamoxifen solution, without the need for gavage, reliably induces CreERT2 activation using a range of expression drivers. While administration of tamoxifen in medicated feed may be the least invasive method of administering tamoxifen (Andersson, Winer, Mork, Molkentin, & Jaisser, 2010), it has various drawbacks for developmental biology research. Embryos will not implant if tamoxifen exposures starts soon after mating (Pugh & Sumano, 1982), a change of diet in early gestation is likely to reduce feed intake, and the amount of tamoxifen consumed by each mouse is likely to be highly variable. Pipette‐feeding tamoxifen retains the advantages of parenteral administration including temporally restricted induction of recombination and controllable dose administration. This approach may also be beneficial for prolonged administrations of tamoxifen to adult animals. Departmental colleagues have found it suitable for studies requiring tamoxifen administration to delete a glomerular gene in the adult kidney. In addition, we have found pipette‐feeding tamoxifen particularly beneficial when gene recombination is required in very young pups.

This study is not intended to compare CreERT2 activation between oral and parenteral tamoxifen administration. The degree of recombination achieved needs to be validated by each group using this technique in their specific tissue of interest. For example, here we show that activation of two NMP‐targeting CreERT2 lines only label a subset of neuroepithelial cells, but this subset is comparable between the two drivers. Equally, the extensive level of recombination achievable following oral administration is shown by efficient Cre‐drivers, such as *Sox2* ^*CreERT2*^ in the closed neural tube. Tissue vascularity and metabolism, as well as the magnitude and specificity of CreERT2 expression, may influence recombination efficiency in different contexts (Heffner et al., 2012; Klinger, Chmura, & Killeen, 2010). In embryos, the timing of tamoxifen administration is also critical. In this study, as in previous reports (Ved et al., 2019), tamoxifen administration at earlier developmental stages was associated with greater embryo toxicity. Pregnancy loss can sometimes be reduced by co‐injection with progesterone (Lizen et al., 2015). Oral administration of progesterone (e.g., in a bioavailable micronized form) was not attempted here as tamoxifen did not change the proportion of dams found to be pregnant at sacrifice. Administration of a high dose of tamoxifen at E8.5 minimally impacted embryo development to E11.5 and did not diminish progression of neural tube closure. This contrasts with a previous study in which tamoxifen administration by SC injection or gavage on E5.5 caused neural tube defects in Cre‐negative embryos (Ved et al., 2019).

The period of neural tube closure is amenable to experimentation ex vivo in whole embryo culture. CreERT2 activation in whole embryo culture with brief OHT treatment enables experiments that require temporally restricted recombination. Examples include studies testing mRNA or protein stability and lineage‐tracing studies avoiding the potential for residual circulating OHT to continue activating CreERT2. In a previous study, OHT was detectable in mouse brains 48 h after a single IP injection of tamoxifen (Jahn et al., 2018). The validation data presented here confirms that *Nkx1.2* ^*CreERT2*^ continues to lineage‐trace neuroepithelial cells 24 h after removal of OHT. TAT‐Cre injection into neurulation‐stage mouse embryos in culture similarly renders novel experimental strategies feasible, such as targeting recombination to a tissue region not demarcated by a promoter construct. Mouse embryos continue to grow in whole embryo culture from the start of neurulation at E8.5 to its completion at E10.5 (Culshaw et al., 2019; Hughes, Greene, Copp, & Galea, 2018; Tung & Winn, 2019). TAT‐Cre can therefore be applied to study neural tube closure, somitogenesis, limb bud initiation, neural crest emigration, cardiac looping, and other processes. This approach will also reduce animal use as it allows animals homozygous for conditional alleles to be bred without requiring the additional breeding generations normally needed to introduce Cre alleles. The main limitation to applying embryo culture techniques is that they can only be used for short‐term studies using currently available methodologies. Advances in bioreactor and live‐imaging technologies are expected to increase their applications in the near future (McDole et al., 2018).

In summary, pipette feeding of tamoxifen to pregnant female mice robustly induces CreERT2 recombination in their embryos without requiring welfare‐compromising oil injections or gavage. Future studies should assess applicability of this route prior to selecting more invasive tamoxifen administration methods. Further temporal and spatial control can be achieved in cultured neurulation stage embryos using ex vivo OHT administration or TAT‐Cre injection. It is anticipated that these approaches will be refined further through the application of tamoxifen caging (Faal et al., 2015; Lu et al., 2012) and optogenetic (Kawano, Okazaki, Yazawa, & Sato, 2016) approaches.

## CONFLICT OF INTEREST

Andrew Copp acts as paid consultant for ViiV Healthcare Limited, with fees going to support his research program. The other authors declare no conflicts of interest.

## AUTHOR CONTRIBUTIONS

D.S., E.M., L.C. and G.L.G. performed and analyzed experiments. G.L.G., N.D.E.G., and A.J.C. provided funding and materials. G.L.G. is responsible for data curation. All authors contributed to writing the manuscript and approved the final version.

## Data Availability

The data that support the findings of this study are available from the corresponding author upon reasonable request.
